# A Paired-Marker Framework for Interpreting Anti-*Ascaris lumbricoides* IgG Serology and Copromicroscopy: A Cross-Sectional Study in Almaty and the Almaty Region, Kazakhstan

**DOI:** 10.3390/pathogens15090896

**Published:** 2026-08-25

**Authors:** Aidar Namet, Mukhit Orynbayev, Timur Davlyatshin, Kulyaisan Sultankulova, Asylzhan Myrzakhmet, Sabyrkhan Barmak, Dulat Inkarbekov, Oralkhan Tusipkanuly, Aiganym Tussipova, Berik Khairullin

**Affiliations:** 1 Scientific Research and Production Center «MVA GROUP» LLP, 1124, Accounting Quarter 096, Koksay Village, Irgeli Rural District, Karasay District, Almaty Region, Almaty 040921, Kazakhstan; ainamet@mail.ru (A.N.); khirullin@mail.ru (B.K.); 2 LLP «Research Institute for Biological Safety Problems» JSC «QazBioPharm» of the Ministry of Health of the Republic of Kazakhstan, Zhambyl Region, Gvardeysky, Astana 080409, Kazakhstan

**Keywords:** *Ascaris lumbricoides*, ascariasis, seroepidemiology, immunological exposure, copromicroscopy, enzyme-linked immunosorbent assay, analytical agreement, Kazakhstan

## Abstract

Background. Stool microscopy and anti-*Ascaris* IgG serology assess different, but related biological domains. Joint interpretation can identify immunological exposure and intestinal egg shedding without assuming one or both markers are a measure of current infection independently. Objective. To develop a four-pattern paired-marker framework for anti-*A. lumbricoides* IgG and copromicroscopy results from adults in Kazakhstan and to assess the qualitative analytical concordance of two IgG enzyme-linked immunosorbent assays (ELISAs). Methods. The cross-sectional field study involved 1000 adult volunteers from Almaty and the region providing matched serum and stool samples. Sera were tested by MVA Group indirect anti-*Ascaris* IgG ELISA, and stool samples were examined by the Kato–Katz and Fülleborn methods. The binary outcome was classified into four pair patterns. All sera were further tested with a commercial ELISA (DRG), and qualitative concordance was calculated. Results. Anti-*Ascaris* IgG was found in 331 of the 1000 subjects (33.1%; 95% CI, 30.3–36.1%), while *A. lumbricoides* eggs were detected in 240 subjects (24.0%; 95% CI, 21.5–26.7%). The paired patterns were IgG-positive/egg-positive in 185 participants (18.5%), IgG-positive/egg-negative in 146 (14.6%), IgG-negative/egg-positive in 55 (5.5%), and IgG-negative/egg-negative in 614 (61.4%). IgG positivity was detected in 77.1% of subjects with detected eggs and 19.2% of subjects without detected eggs (odds ratio, 14.15; 95% CI, 9.96–20.09; *p* < 0.001). The MVA Group and DRG ELISAs agreed for 990 of 1000 specimens (99.0%; 95% CI, 98.2–99.5%), with positive and negative agreements of 98.2% and 99.4%, respectively (κ = 0.98). Conclusions. Serology and copromicroscopy showed a high association, but they are not interchangeable. The paired-marker paradigm clarified concordant and discordant patterns while retaining IgG as a marker of past or ongoing immune exposure and stool eggs as markers of shedding at the time of sampling. High inter-assay agreement suggests analytical consistency within this serum panel, but does not imply diagnostic accuracy for active ascariasis.

## 1. Introduction

Important advances in surveillance, sanitation, and preventive treatment have not eradicated soil-transmitted helminth infections as a major public health problem. The major human soil-transmitted helminths are *Ascaris lumbricoides*, *Trichuris trichiura*, and hookworms. Sanitation, water and food hygiene, poverty, environmental conditions, and access to control programs are important determinants of their distribution [1,2,3,4,5,6]. Recent global burden assessments indicate that transmission and associated disability have decreased in many settings, but remain unevenly distributed, with continuing gaps in surveillance and control [2,3]. Central Asia has been identified as a region in which neglected parasitic diseases are poorly represented in the global literature and where the evidence available for program planning has traditionally been fragmented [7].

Transmission of *A. lumbricoides* occurs through the ingestion of embryonated eggs from environments contaminated with human feces. Adult worms live in the intestine, and diagnosis is usually based on the detection of eggs in feces [1,3]. Copromicroscopy using the Kato–Katz thick-smear technique is important for epidemiological mapping and program monitoring and remains relevant to the World Health Organization’s diagnostic target product profile for soil-transmitted helminth surveillance [8,9]. Egg detection has particular biological significance as a marker of intestinal egg shedding at the time of examination. However, its diagnostic yield depends on worm patency, infection intensity, temporal variation in egg production, the number and quantity of specimens examined, and laboratory procedures. Reviews and latent-class meta-analyses have shown that the sensitivity of copromicroscopic approaches decreases in low-intensity settings, but improves when additional slides or repeated stool samples are examined [10,11,12]. Molecular assays can detect more low-intensity infections, but their routine use is limited by cost, infrastructure requirements, variation in protocols, and the absence of a perfect reference standard [10,13].

Serological surveillance provides a complementary area of measurement. Antibody assays can detect host responses following exposure, even when examination of a single stool sample does not reveal eggs. Serological surveys have provided useful information about community exposure and transmission for a range of infectious diseases [14,15,16]. However, antibody detection in helminth infections requires careful interpretation. IgG may persist after infection has resolved, may result from exposure that does not culminate in patent intestinal infection, and may be influenced by recurrent exposure, antigen composition, immunoglobulin isotype or subclass, individual immune responsiveness, and cross-reactivity with other helminths [17,18,19]. Anti-*Ascaris* IgG seropositivity should therefore be interpreted as evidence of previous or ongoing immunological exposure and not as proof of active intestinal ascariasis per se.

More recent studies of *Ascaris* have demonstrated both the benefits and limitations of serology. In Ethiopia, serological responses to specific antigens were examined alongside copromicroscopy, revealing age- and treatment-related differences at the population level [20,21]. Longitudinal follow-up also indicated that antibody responses and recurrent egg detection may occur within the same populations, but do not necessarily coincide in the same individuals [22]. Among schoolchildren in western Kenya, IgG responses, particularly IgG1 responses to adult-worm excretory–secretory products, showed promise as complements to microscopy and quantitative PCR [23]. These findings support the further development of antibody-based surveillance, but also demonstrate that performance varies according to the antigen, isotype, population, and reference definition. A recent scoping review identified substantial variability among serological assays for human soil-transmitted helminth infections and concluded that their large-scale validation and integration into control programs remain limited [18].

Discrepancies between antibody and stool findings are difficult to interpret and are often obscured when studies report only separate prevalence estimates or use one method as a proxy reference for the other. At the individual level, four paired-testing patterns can be distinguished: IgG-positive/copromicroscopy-positive, IgG-positive/copromicroscopy-negative, IgG-negative/copromicroscopy-positive, and IgG-negative/copromicroscopy-negative. No single mechanism necessarily explains either discordant pattern. Antibody persistence, prepatent exposure, sporadic or absent egg production, low-intensity infection, variability in host immune responses, cross-reactivity, and test thresholds may all contribute to discordance between serological and copromicroscopic findings [10,17,18,19,20,21,22,23]. Thus, an operational paired-marker approach can provide a more transparent epidemiological characterization by preserving the biological significance and limitations of each marker rather than forcing potentially ambiguous findings into dichotomous categories of true and false infection.

A major methodological difficulty is the comparison of serological platforms. An agreement study between a novel assay and a commercial comparator can determine whether both methods consistently classify the same panel of sera, but it cannot establish diagnostic sensitivity or specificity without independent verification of infection status. Therefore, positive and negative percentage agreement, chance-corrected agreement, and discordant-pair analysis are more appropriate than terminology based on diagnostic accuracy when neither antibody assay serves as a reference standard [10,18]. This distinction is important when evaluating locally developed assays: analytical comparability is valuable, but should not be interpreted as complete clinical validation or as confirmation that seropositivity indicates current infection.

The present cross-sectional field study was designed to implement a structured paired-marker framework for analyzing anti-*A. lumbricoides* IgG and copromicroscopy data from adult volunteers living in Almaty and the surrounding region. The framework categorized each participant into one of four serology–copromicroscopy patterns, with IgG serving as evidence of immunological exposure and the detection of eggs in stool serving as evidence of egg shedding at the time of collection. The secondary objectives were to estimate the positive proportions obtained using each approach, explore demographic and contextual correlates of IgG seropositivity, and evaluate qualitative analytical agreement between the MVA Group anti-*Ascaris* IgG ELISA and a commercial DRG ELISA. The study did not use a diagnostic reference standard. Instead, the commercial assay was used as a comparator to assess paired interpretation and analytical agreement rather than the sensitivity or specificity of the locally developed assay for active ascariasis.

## 2. Materials and Methods

### 2.1. Study Design, Setting, and Methodological Objective

This cross-sectional field study utilized a structured paired-marker framework for the population-level analysis of anti-*Ascaris lumbricoides* IgG findings. The framework consisted of three interconnected components: (i) an indirect ELISA for anti-*A. lumbricoides* IgG, serving as a marker for prior or ongoing immunological exposure; (ii) copromicroscopic identification of *A. lumbricoides* eggs, indicating intestinal egg shedding at the time of sampling; and (iii) a comparative evaluation of qualitative analytical concordance between the MVA Group ELISA and a commercial DRG ELISA. The primary methodological aim was to assess how paired serological and copromicroscopic findings might be interpreted without considering IgG seropositivity as a proxy for active ascariasis. The estimation of prevalence and exploratory studies of contextual variables served as supplementary applications of the methodology.

The study was conducted from August 2025 to June 2026 among adults residing in Almaty and the Almaty region, southeastern Kazakhstan (approximately 43.9368° N, 76.8260° E). Urban areas were characterized by a predominantly built environment and centralized municipal services, whereas rural areas comprised administratively designated villages where household land plots and agricultural activity were more common. Residence was classified according to the official administrative status of the participant’s reported place of residence. Serum and stool samples were obtained from each participant and associated at the individual level. The report was compiled in accordance with the Strengthening the Reporting of Observational Studies in Epidemiology (STROBE) guidelines [24].

### 2.2. Study Population, Recruitment, and Eligibility

Adult volunteers were recruited in Almaty and the Almaty region during 2025–2026. The study protocol predetermined a total sample of 1000 participants, with 500 participants recruited during each study year. The statistical adequacy of this target was assessed using the formula for estimating a single proportion, (*n* = Z^2^*p*(1 − *p*)/d^2^). Because no reliable previous estimate was available for adults in the study area, an expected proportion of 50% was used, together with a 95% confidence level and 5% absolute precision. The calculated minimum sample size was 385 participants. Therefore, the protocol-defined target of 1000 participants exceeded the minimum required sample size. Consecutive recruitment continued until 500 eligible participants with complete matched serum and stool results had been included in each study year.

Before any study procedure, each potential participant received information about the study and signed written informed consent. Participants then completed the volunteer questionnaire and provided venous blood and stool samples. Stool samples could be returned on the day of enrollment or the following day. Each participant was assigned a unique identification number, which was used to link the questionnaire data with the corresponding serum and stool samples.

The inclusion criteria were age ≥18 years; residence in Almaty or the Almaty region; signed written informed consent; agreement to complete the questionnaire and provide venous blood and stool samples; no anthelmintic treatment during the previous three months; and availability of a complete questionnaire and suitable paired serum and stool samples. The exclusion criteria were age <18 years; inability or refusal to provide informed consent; contraindication to venipuncture; intoxication at the time of enrollment; anthelmintic treatment during the previous three months; withdrawal from the study; an incomplete questionnaire; or an unsuitable or incomplete set of serum and stool samples. No restrictions were applied based on sex or ethnicity.

### 2.3. Questionnaire and Contextual Variables

At enrollment, participants completed a standardized program-wide questionnaire covering demographic characteristics, residence, household conditions, contact with soil and domestic animals, access to gardens or land plots, drinking water sources, fishing and hunting activities, consumption of raw or undercooked meat, and food-handling practices such as washing fruit and vegetables. Because the questionnaire was developed for a broader epidemiological monitoring program covering several parasitic infections, it included variables that were not specific transmission pathways for ascariasis. All variables were examined as exploratory factors and were not interpreted as diagnostic indicators or evidence of causal relationships.

Age was reported in completed years and grouped before analysis into the following categories: 18–24, 25–44, 45–59, 60–74, and 75–89 years. No participants were aged ≥90 years. Residence was categorized as urban or rural based on the participant’s reported place of residence. Questionnaire data were linked to laboratory data using a unique research identification number and checked for completeness and internal consistency before analysis.

### 2.4. Collection, Identification, and Handling of Specimens

All participants provided venous blood and stool samples. Qualified staff collected 5–7 mL of venous blood into a single-use vacuum tube containing a clot activator. The blood was allowed to clot for 20–30 min and then centrifuged at 1500–2000× *g* for 10 min. Sera were transferred into labeled tubes, divided into aliquots of at least 0.5 mL when sufficient volume was available, and stored at −20 °C until analysis. Serum samples with insufficient volume, marked hemolysis, lipemia, or visible contamination were excluded.

Participants collected stool samples in disposable, leak-proof, sterile containers according to the instructions provided by the study personnel. Samples were transported to the laboratory at 2–8 °C and examined within 24 h of collection. Each stool container was labeled with the same unique research identification number as the corresponding serum sample. The matched identifiers were verified before the laboratory results were entered into the study database.

### 2.5. Enzyme-Linked Immunosorbent Assay for Anti-Ascaris IgG (MVA GROUP)

Sera were tested by the Ascarida-IgG-Human-ELISA–MVA Group test system (Research and Production Center MVA Group LLP, Almaty, Kazakhstan), an indirect ELISA method with native excretory–secretory antigen of *A. lumbricoides* fixed on polystyrene microplate wells. The assay was employed as a serological research tool for the assessment of anti-*A. lumbricoides* IgG, and a positive result was not considered indicative of a current intestinal infection.

Samples were analyzed in duplicate, with 20 µL of serum and 80 µL of serum diluent added to each well to obtain a 1:5 dilution. The plates were incubated for 30 min at 37 °C, after which the wells were washed five times with phosphate-buffered saline containing Tween 20 (PBS-T). One hundred microliters of horseradish peroxidase-conjugated anti-human IgG was added, and a second 30 min incubation was performed at 37 °C, followed by five washes. One hundred microliters of tetramethylbenzidine substrate was added and incubated for 15 min at 18–25 °C in the absence of light. The reaction was quenched by adding 100 µL of 0.9 M sulfuric acid. The optical density (OD) was measured at 450 nm with the reference at 630 nm.

Each run included a reagent blank, negative controls, and positive controls. A run was considered acceptable if the negative-control optical density (OD) was ≤0.20, the positive-control OD was ≥1.0, and the background and duplicate performance met laboratory quality-control standards. Where the duplicate coefficient of variation was ≥15%, the average optical density of the duplicates was used, and samples above this limit were reanalyzed. The run-specific critical optical density (OD) was determined as ODcrit = average OD of negative control +0.200. Samples with a mean OD ≥ ODcrit were considered positive, and samples with a mean OD < ODcrit were considered non-positive. This binary criterion was applied consistently to the paired analyses and the prevalence analyses.

### 2.6. Copromicroscopic Examination

Stool specimens were examined using the complementary Kato–Katz thick-smear [25] and Fülleborn flotation techniques [26]. Kato–Katz smears were prepared and examined by light microscopy according to standard procedures. Fülleborn flotation was included as a complementary qualitative concentration method because the underlying saturated-brine flotation principle can increase the recovery of *A. lumbricoides* eggs from human stool specimens [27]. For this technique, stool was homogenized in saturated sodium chloride solution, followed by flotation and microscopic examination. The Fülleborn technique was not used to quantify egg burden or as an independent reference standard.

Helminth eggs were identified by trained laboratory parasitologists based on their morphological characteristics, including size, shape, shell structure, external surface, and internal contents, in accordance with the World Health Organization bench aids for the diagnosis of intestinal parasites [25]. Although eggs of other helminths were occasionally observed, the predefined outcome for the present analysis was the detection of eggs morphologically compatible with *A. lumbricoides*.

A specimen was classified as copromicroscopy-positive for *A. lumbricoides* when at least one morphologically compatible egg was detected by either technique and as copromicroscopy-negative when no compatible eggs were detected by either technique. This outcome represented egg shedding at the time of examination and was not considered a perfectly sensitive reference standard for infection.

### 2.7. The Operational Significance of Paired Markers

The main methodological analysis cross-classified the binary MVA Group IgG outcome with the binary copromicroscopy outcome at the participant level. Four mutually exclusive patterns were identified: IgG-positive/copromicroscopy-positive, IgG-positive/copromicroscopy-negative, IgG-negative/copromicroscopy-positive, and IgG-negative/copromicroscopy-negative. These designations are descriptive of observable trends in the laboratory, not discrete clinical situations. Contradictory findings were not ascribed to a single biological explanation, and persistence of antibodies, timing and intensity of exposure, intermittent or low-intensity egg shedding, and variability in specimens and characteristics of the assay may have played a role.

To differentiate between the two measurement domains, we applied a paired framework: IgG seropositivity was interpreted as a marker for immunological exposure, while egg detection was interpreted as evidence of intestinal egg shedding at the time of sampling. The other was not marked for redefinition.

### 2.8. Analytical Concordance to DRG ELISA

All 1000 serum specimens were tested by the *Ascaris lumbricoides* IgG ELISA/EIA (catalog no. EIA-3817; DRG Instruments GmbH, Marburg, Germany) according to the manufacturer’s instructions. The DRG results were interpreted based on the manufacturer’s qualitative standards. For the binary comparison, non-positive included negative and equivocal results where applicable. The DRG assay was used as a commercial comparator, but not as a diagnostic reference standard.

The paired MVA Group and DRG classifications were tabulated in a 2 × 2 table. Overall percentage agreement was the proportion of specimens classified consistently. Positive percentage agreement was the proportion of DRG-positive specimens that were identified as positive by MVA Group, and negative percentage agreement was the proportion of DRG-non-positive specimens that were identified as non-positive by MVA Group. Chance-adjusted concordance was assessed by Cohen’s kappa. Directional asymmetry of discordant pairs was assessed by exact McNemar testing. Sensitivity, specificity, and predictive values were not calculated, as true serological status was not established independently.

### 2.9. Statistical Analysis

The main methodological result was the distribution of individuals in the four paired serology–copromicroscopy patterns. Secondary descriptive outcomes were the proportion positive for MVA Group IgG ELISA and copromicroscopy. Categorical variables are presented as numbers and percentages. Binomial proportions presented as two–sided 95% Wilson confidence intervals. The analytical sample included only those with paired laboratory and questionnaire data, so no imputation was required.

Differences in IgG positivity by age group, sex, and residency category were evaluated using Pearson’s χ^2^ test or Fisher’s exact test in cases of low expected cell counts. We correlated copromicroscopy positivity with IgG positivity using paired 2 × 2 tables to calculate an odds ratio with 95% confidence intervals.

Exploratory correlations between IgG positivity and the contextual questionnaire factors described in the Results section were determined through logistic regression. The multivariable model included sex, domicile, interaction with domestic animals, access to gardens or private homes, fishing, hunting, tap water, open water, eating raw or undercooked meat, and eating inadequately washed vegetables or fruit. Results are expressed as univariable and adjusted odds ratios with 95% confidence intervals (CIs). The analyses were viewed as relationships within the sample of volunteers, not as causal or representative of the population.

For the MVA Group–DRG comparison, Cohen’s kappa and overall, positive, and negative percentage agreement were calculated from paired qualitative data, and discordant pairings were analyzed with the exact two-sided McNemar test [28]. All hypothesis tests were two-sided and conducted at a nominal significance level of *p* < 0.05.

### 2.10. Ethical Considerations

The study was conducted in accordance with the Declaration of Helsinki [29] and national regulations for biomedical studies involving human subjects. The protocol was approved by the Bioethics Committee of the Research and Production Center of MVA Group LLP (Protocol No. 1, 8 January 2025) and the Ethics Committee of the Association of Bioethics and Medical Law (Meeting No. 20, 8 January 2025).

All participants were informed about the study procedures, specimen collection, intended use of the data, confidentiality, and voluntary nature of participation. Written informed consent was obtained before the start of any study procedure. The questionnaire data and specimens were anonymized, and the analysis was performed on the coded dataset, with results presented in aggregate form.

## 3. Results

### 3.1. Study Sample

The analytical sample comprised 1000 adult volunteers, all of whom completed the questionnaire and provided paired serum and stool specimens. Participants were 18–82 years of age. Most participants were women (70.2%) and lived in urban areas (68.7%). The largest age group was 25–44 years (Table 1).

### 3.2. Serological and Copromicroscopic Outcomes

The serological and copromicroscopic findings are summarized in Table 2. The proportion of participants with anti-*Ascaris lumbricoides* IgG positivity exceeded the proportion with copromicroscopic egg detection by 9.1 percentage points.

### 3.3. Paired Serology–Copromicroscopy Patterns

The paired distribution included both concordant and discordant results (Table 3). Among participants with eggs detected, 77.1% (185/240) were IgG-positive compared with 19.2% (146/760) among those without detected eggs (OR 14.15; 95% CI 9.96–20.09; *p* < 0.001).

### 3.4. IgG Positivity by Age, Sex, and Residence

IgG positivity did not differ significantly among the revised age groups (*p* = 0.354). Positivity was higher among women than men (*p* = 0.008), whereas no significant difference was observed between urban and rural participants (*p* = 0.653) (Table 4).

### 3.5. Contextual Variables Associated with IgG Positivity

In univariable analyses, female sex was associated with higher odds of IgG positivity, whereas fishing and consumption of raw or undercooked meat were associated with lower odds. Insufficiently washed vegetables or fruit were not significantly associated with IgG positivity. After adjustment, only consumption of raw or undercooked meat remained significant and was associated with lower odds of IgG positivity (aOR 0.60; 95% CI 0.45–0.79; *p* < 0.001). Female sex, insufficiently washed vegetables or fruit, and fishing were not significant in the adjusted model (Table 5).

### 3.6. Agreement Between Copromicroscopic Methods

Of the 240 stool specimens positive by either copromicroscopic method, 193 (80.4%) were positive by Kato–Katz and 146 (60.8%) by Fülleborn. Both methods produced the same result for 859/1000 specimens, corresponding to an overall agreement of 85.9% (95% CI: 83.6–87.9%) and Cohen’s κ of 0.50. Among the discordant specimens, 94 were positive only by Kato–Katz and 47 only by Fülleborn. The difference between the discordant results was statistically significant (exact McNemar *p* < 0.001) (Table 6).

Kato–Katz was used as the primary comparator for calculating positive and negative percentage agreement. The agreement measures should not be interpreted as diagnostic sensitivity or specificity. CIs for percentage agreement were calculated using the Wilson method.

### 3.7. Analytical Agreement Between the MVA Group and DRG ELISAs

The MVA Group and DRG ELISAs showed high qualitative agreement (Table 7). The 10 discordant pairs showed no evidence of directional imbalance by exact McNemar testing (*p* = 0.754).

## 4. Discussion

The findings from the Almaty sample show why the study is most informative when the serological and stool results are examined together. Anti-*Ascaris lumbricoides* IgG was strongly associated with detection of morphologically compatible eggs, supporting the biological relevance of the antibody response measured by the study assay. At the same time, discordant result combinations occurred in both directions. Paired analysis distinguished participants with concurrent antibody detection and egg shedding from those with only one of these findings, providing a more informative epidemiological description than either positive proportion considered alone.

The positive findings in this adult sample are also relevant to the regional epidemiology of ascariasis. Evidence from Central Asia remains limited, and adults are less often included in soil-transmitted helminth surveys than school-aged children [7]. In Jimma, Ethiopia, Dana et al. found that *Ascaris* egg positivity decreased with age, whereas IgG4 responses to larval and adult-worm antigens remained detectable, and for one antigen increased across age groups [20]. Vlaminck et al. subsequently showed that community IgG4 rates declined in parallel with egg output after mass drug administration, supporting the use of antibody responses to monitor changes at the population level [21]. In the present study, total IgG positivity did not differ significantly among the adult age groups. Direct numerical comparison is not appropriate because the studies used different antigens, antibody classes, stool methods, and populations. Their results nevertheless point to the same practical issue: restricting surveillance to children may overlook substantial exposure among adults. The Almaty findings extend this evidence to a setting for which paired adult data have been scarce.

The strong association between the two laboratory findings is important. IgG was detected in 77.1% of participants with eggs and in 19.2% of those without eggs, producing an odds ratio of 14.15. This shows that IgG positivity was much more frequent when egg shedding was present. The IgG-positive/egg-negative group was nevertheless larger than the egg-positive/IgG-negative group (146 versus 55 participants). A similar imbalance has been reported in longitudinal research. Dana et al. found that cumulative IgG4 positivity exceeded cumulative egg detection and that baseline IgG4 levels were associated with subsequent egg counts and repeated positive stool examinations [22]. Mugo et al. also showed that Kato–Katz and Mini-FLOTAC detected fewer *Ascaris* infections than quantitative PCR in Kenyan schoolchildren [23]. These studies support two reasonable interpretations of the larger IgG-positive/egg-negative group: antibodies may remain detectable after earlier exposure, and a single stool examination may fail to detect low or variable egg output. The smaller egg-positive/IgG-negative group may reflect weak antibody responses, antibody concentrations below the assay cutoff, or differences between total IgG and the IgG subclasses that respond most strongly to particular antigens. At a wider program level, Roose et al. found that serological estimates exceeded stool-based estimates in most Ethiopian districts and that applying stool-based thresholds directly to serology produced only moderate agreement in control classifications [30]. The present study did not evaluate treatment thresholds, but the comparison shows why the 9.1-percentage-point difference observed in Almaty should not be converted into 91 additional current intestinal infections. The individual paired results, rather than the difference between the two overall percentages, provide the sounder interpretation.

The demographic results do not identify a clearly defined adult group in which IgG positivity was concentrated. Positivity was found across all age groups, without a statistically significant difference. IgG positivity was higher among women than among men in the unadjusted analysis. However, when sex was included with residence and the questionnaire variables in the multivariable logistic regression model, it was no longer statistically significant. Female sex should therefore not be described as an independent risk factor. Urban and rural residence showed no significant association with IgG positivity either. This does not demonstrate that transmission conditions are identical in urban and rural areas. It indicates only that the broad residence category used in this study did not separate participants according to IgG status. Food distribution, occupational or household soil contact, movement between urban and rural areas, and previous residence may cross this simple geographic division.

The questionnaire analysis likewise provided little evidence that a single reported behavior explained IgG positivity. In the multivariable logistic regression model, contact with domestic animals, access to a garden or land plot, fishing, hunting, water source, residence, and sex were not statistically significant. Insufficient washing of vegetables or fruit showed an association in the expected direction, but the evidence was not sufficient to identify it as a risk factor (aOR 1.41; 95% CI 0.96–2.05; *p* = 0.076). This result is compatible with fecal–oral transmission, but remains inconclusive. By contrast, raw or undercooked meat consumption was associated with lower odds of IgG positivity. Because meat is not a route of *A. lumbricoides* transmission, this association must not be interpreted as protective. It may represent other differences in diet, lifestyle, or socioeconomic conditions that were not measured, or it may be a chance finding within an exploratory analysis. The absence of associations with the other questionnaire variables does not contradict the established importance of water, sanitation, hygiene, and safe food handling [5,6]. Those studies examined more direct measures of sanitation and hygiene, whereas the present questionnaire recorded current self-reported practices and the outcome was an antibody response that may reflect earlier exposure. The most defensible interpretation is that no single variable measured here adequately represented the conditions that produced exposure.

The comparison of the MVA Group and DRG ELISAs produced the clearest analytical result. The assays agreed for 990 of 1000 sera, with 99.0% overall agreement, 98.2% positive percentage agreement, 99.4% negative percentage agreement, and a Cohen’s κ of 0.98. The ten discordant results were nearly evenly divided between the two directions, which argues against a systematic tendency of either assay to classify more samples as positive. Testing the complete serum set rather than a selected group of positive and negative samples strengthens this comparison. The use of adult-worm excretory–secretory antigen in the MVA Group assay is also supported by Mugo et al., who found that IgG1 responses to adult *Ascaris* excretory–secretory products had the best performance among the antibody responses they evaluated [23]. Their work involved children, subclass-specific quantitative measurements, and a reference definition that included qPCR, so its performance estimates cannot be applied directly to the present total-IgG assay. It does, however, support the choice of antigen. Because the DRG ELISA was a comparator rather than an independent standard for current intestinal ascariasis, the present study estimates agreement, not diagnostic sensitivity or specificity. A recent review found wide variation in the antigens, immunoglobulin classes, cutoffs, and validation procedures used in serological studies of soil-transmitted helminths [18]. Within that diverse field, agreement of 99.0% across 1000 paired tests is a strong basis for further evaluation of the MVA Group assay using external serum panels, samples from individuals with other helminth infections, and reproducibility testing across laboratories and reagent batches.

The study is strengthened by complete matching of serum and stool data for all participants, the use of two copromicroscopic methods, and testing of every serum sample with both ELISAs. These features support the internal comparison of the laboratory findings. The main limitations concern the questions that cannot be answered from this design. Consecutive recruitment of volunteers prevents direct extrapolation of the positive proportions to the wider population of Almaty or Kazakhstan. Examination of one stool specimen and the absence of qPCR limit resolution of the different serology–stool combinations, while the cross-sectional design does not establish when exposure occurred. The effect of specimen number and infection intensity on stool-based detection has been demonstrated in repeated-sampling studies, meta-analyses, and comparisons with molecular methods [9,12,13,31]. These limitations should guide interpretation, but they do not alter the observed association between IgG and egg detection or the measured agreement between the ELISAs.

For epidemiological monitoring in Kazakhstan, the results support the inclusion of adults and the use of matched serological and stool data when both exposure and egg shedding are of interest. They also provide a clear basis for continued evaluation of the MVA Group ELISA. Representative regional sampling, repeated stool examinations, molecular testing, and external assay validation would extend the present work from a well-defined field comparison to broader population surveillance and diagnostic evaluation.

## 5. Conclusions

In this adult volunteer group from Almaty and its vicinity, anti-*Ascaris* IgG seropositivity was more prevalent than the copromicroscopic detection of eggs (33.1% vs. 24.0%), and paired testing showed both significant concordance and discordance in both directions. The results indicate that the two indicators are correlated, but not interchangeable. Seropositivity should be regarded as evidence of past or current immunological exposure, while a positive stool examination indicates egg shedding at the time of examination. The four observed cross-classification patterns provide a simple way to make this distinction without assigning ambiguous outcomes to assumed true or false infection states.

The MVA Group and DRG ELISAs were 99.0% qualitatively concordant, indicating analytical consistency for this serum panel, but not diagnostic sensitivity, specificity, or equivalency for active ascariasis. Recruitment was on a voluntary basis, and the methodology was cross-sectional. Therefore, the numerical estimates refer to the recruited sample and should not be generalized to the population prevalence. Large multicenter studies with serial stool examination, quantitative egg counts, molecular detection, longitudinal serology, and external validation of the assays are needed to clarify discordant patterns and the appropriate surveillance role of anti-*Ascaris* IgG.

## Figures and Tables

**Table 1:** Characteristics of the analytical sample and paired-data availability.

Characteristic	*n*	%
Total participants	1000	100.0
Paired serum–stool data available	1000	100.0
Sex		
Women	702	70.2
Men	298	29.8
Age group		
18–24 years	144	14.4
25–44 years	444	44.4
45–59 years	245	24.5
60–74 years	154	15.4
75–89 years	13	1.3
Residence		
Urban	687	68.7
Rural	313	31.3

Percentages use N = 1000 as the denominator.

**Table 2:** Anti-*Ascaris lumbricoides* IgG and copromicroscopy outcomes.

Laboratory Outcome	Positive, *n*/*N*	Positive, %	95% CI
Anti-*A. lumbricoides* IgG ELISA	331/1000	33.1	30.3–36.1
Copromicroscopy for *A. lumbricoides* eggs	240/1000	24.0	21.5–26.7

CI, Wilson 95% confidence interval. The MVA Group ELISA result is classified as positive or non-positive.

**Table 3:** Paired anti-*Ascaris* IgG serology and copromicroscopy results.

Igg Result	Eggs Detected, *n* (%)	No Eggs Detected, *n* (%)	Total, *n* (%)
Positive	185 (18.5)	146 (14.6)	331 (33.1)
Non-positive	55 (5.5)	614 (61.4)	669 (66.9)
Total	240 (24.0)	760 (76.0)	1000 (100.0)

Values are n (% of the total analytical sample, N = 1000). Egg detection by either copromicroscopic technique was classified as positive.

**Table 4:** Anti-*Ascaris* IgG positivity by age, sex, and residence.

Characteristic	Total, *n*	IgG-Positive, *n*	Positive, %	95% CI	Group *p* Value
Age group					0.354
18–24 years	144	39	27.1	20.5–34.9	—
25–44 years	444	144	32.4	28.2–36.9	—
45–59 years	245	91	37.1	31.3–43.3	—
60–74 years	154	53	34.4	27.4–42.2	—
75–89 years	13	4	30.8	12.7–57.6	—
Sex					0.008
Women	702	251	35.8	32.3–39.4	—
Men	298	80	26.8	22.1–32.1	—
Residence					0.653
Urban	687	231	33.6	30.2–37.2	—
Rural	313	100	31.9	27.0–37.3	—

—, not applicable; *p* values refer to the overall comparison within each characteristic.

**Table 5:** Contextual variables associated with anti-*Ascaris* IgG positivity.

Factor	IgG-Positive, *n*/*N*	Positive, %	Univariable OR (95% CI)	Adjusted OR (95% CI)	Adjusted *p* Value
Female sex	251/702	35.8	1.52 (1.12–2.05)	1.33 (0.96–1.84)	0.084
Rural residence	100/313	31.9	0.93 (0.70–1.23)	0.87 (0.61–1.22)	0.412
Any domestic animals	138/456	30.3	0.79 (0.60–1.03)	0.89 (0.67–1.17)	0.399
Garden/private house	60/189	31.7	0.93 (0.66–1.30)	1.07 (0.71–1.61)	0.739
Fishing	32/138	23.2	0.57 (0.37–0.86)	0.69 (0.42–1.16)	0.164
Hunting	16/63	25.4	0.67 (0.38–1.20)	1.08 (0.54–2.17)	0.826
Tap water use	283/867	32.6	0.86 (0.59–1.26)	1.04 (0.69–1.55)	0.867
Open water use	73/231	31.6	0.92 (0.67–1.25)	1.10 (0.78–1.54)	0.587
Raw/undercooked meat	162/578	28.0	0.58 (0.45–0.76)	0.60 (0.45–0.79)	<0.001
Insufficiently washed vegetables/fruit	56/148	37.8	1.28 (0.89–1.83)	1.41 (0.96–2.05)	0.076

**Table 6:** Agreement between Kato–Katz and Fülleborn for the detection of *A. lumbricoides* eggs.

**A. Paired Results**			
Kato–Katz Result	Fülleborn-Positive, *n*	Fülleborn-Negative, *n*	Total, *n*
Positive	99	94	193
Negative	47	760	807
Total	146	854	1000
**B. Agreement Measures**			
Measure	Numerator/denominator	Estimate	95% CI
Overall percentage agreement	859/1000	85.9%	83.6–87.9%
Positive percentage agreement	99/193	51.3%	44.3–58.3%
Negative percentage agreement	760/807	94.2%	92.3–95.6%
Cohen’s κ	—	0.50	—
Exact McNemar *p* value	94 vs. 47 discordant results	<0.001	—

—, not applicable. A 95% CI was not calculated for Cohen’s κ.

**Table 7:** Agreement between the MVA Group and DRG ELISAs.

**A. Paired Qualitative Classifications**			
MVA Group Result	DRG-Positive, *n*	DRG-non-positive, *n*	Total, *n*
MVA positive	327	4	331
MVA non-positive	6	663	669
Total	333	667	1000
**B. Agreement Measures**			
Measure	Numerator/Denominator	Estimate	95% CI
Overall percentage agreement	990/1000	99.0%	98.2–99.5%
Positive percentage agreement	327/333	98.2%	96.1–99.2%
Negative percentage agreement	663/667	99.4%	98.5–99.8%
Cohen’s κ	—	0.98	—
Exact McNemar *p* value	4 vs. 6 discordant pairs	0.754	—

—, not applicable. A 95% CI was not calculated for Cohen’s κ.

## Data Availability

Data are contained within the article.
